# Anti-Phosphatidylethanolamine and Anti-Phosphatidylserine Antibodies—Association with Renal Involvement, Atherosclerosis, Cardiovascular Manifestations, Raynaud Phenomenon and Disease Activity in Polish Patients with Systemic Lupus Erythematosus

**DOI:** 10.3390/biom12101328

**Published:** 2022-09-20

**Authors:** Katarzyna Fischer, Hanna Przepiera-Będzak, Iwona Brzosko, Marcin Sawicki, Anna Walecka, Marek Brzosko

**Affiliations:** 1 Individual Laboratory for Rheumatologic Diagnostics, Pomeranian Medical University in Szczecin, Unii Lubelskiej 1, 71-252 Szczecin, Poland; 2 Department of Rheumatology, Internal Medicine, Geriatrics and Clinical Immunology, Pomeranian Medical University in Szczecin, Unii Lubelskiej 1, 71-252 Szczecin, Poland; 3 Department of Imaging Diagnostics and Interventional Radiology, Pomeranian Medical University in Szczecin, Unii Lubelskiej 1, 71-252 Szczecin, Poland

**Keywords:** anti-phosphatidylethanolamine antibodies, anti-phosphatidylserine antibodies, systemic lupus erythematosus, antiphospholipid syndrome, renal involvement, cardiovascular risk, smoking status

## Abstract

Objective. To evaluate the association between anti-phosphatidylethanolamine (aPE) and anti-phosphatidylserine (aPS) antibodies and cardiovascular risk, organ involvement and disease activity in systemic lupus erythematosus (SLE) patients. Methods. We studied 93 SLE patients and 30 controls. We analyzed levels of anti-phospholipid antibodies, including aPS and aPE, the profiles of antinuclear, anti-neutrophil cytoplasmic (ANCA) and anti-endothelial antibodies, carotid intima-media thickness (cITM) and atherosclerotic plaque presence, ankle-brachial and high resistance indices, atherosclerotic risk factors, organ manifestations and treatment. Results. Levels of aPS and aPE were significantly higher in SLE patients in comparison with the controls (*p* = 0.038 and *p* = 0.044, respectively). aPS was associated with the risk of Raynaud’s phenomenon (*p* = 0.021) development. aPE increased the risk of renal involvement (*p* = 0.049), cerebral stroke (*p* = 0.050), high vlues of cIMT (*p* = 0.041) development as well as occurrence of selected serological markers associated with activity of the disease such as anti-double stranded DNA (*p* = 0.021). The long duration of regular smoking (*p* = 0.021) and the high number of cigarettes/day (*p* = 0.015) were significantly associated with the risk of aPE occurrence. Conclusions. Patients with aPS and aPE are at risk of vascular involvement. Especially the presence of aPE may significantly increase the risk of thrombotic complications development in SLE patients without classical serological markers of APS. Finally, aPE might be used as a marker of disease activity and risk of renal injury development in this patient group. The classical atherosclerotic markers including lipid indices play an important role in complex analysis of cardiovascular risk in lupus patients and enable to identify patients at the highest risk and implement effective preventive, diagnostic and therapeutic procedures.

## 1. Introduction

Systemic lupus erythematosus (SLE) is autoimmune, chronic rheumatic disease characterized by a broad spectrum of clinical manifestations and a wide range of autoantibodies production [1]. The main contributing factors for tissue damage in SLE are autoantibodies and immune complexes deposition. However, pathogenic mechanisms underlying this disease are still unknown and its course and organ involvements are unpredictable [2,3].

In addition to antinuclear antibodies (ANA) positivity in the course of SLE other antibodies are observed such as anti-phospholipid (aPL) and anti-neutrophil cytoplasmic (ANCA). The main targets of aPL are proteins bound to anionic phospholipids located on endothelium and other cellular membranes [4]. In clinical practice, aPL are measured as anticardiolipin (aCL), anti-beta 2 glycoprotein I (aβ2-GPI) antibodies and lupus anticoagulant (LA) test. Persistent aPL positivity, together with thrombotic vascular events, obstetric complications, or both, are the basis for diagnosing the antiphospholipid syndrome (APS) [4]. APS is considered the most prevalent acquired thrombophilia and is found in 20–35% of SLE patients. The potential pathogenic and diagnostic role of non-criteria aPL has been the matter of discussion for many years. Early studies performed in 1990s have already paid attention to aPL directed against other than cardiolipin antigens in SLE. They documented significantly increased levels of selected aPL in lupus patients and described a wide profile of potential antigens [5,6]. However, the clinical significance of most of them has not been clearly assessed. On the contrary, some reports showed increasing evidence of a relationship between the clinical manifestations of APS and antibodies directed against phosphatidylethanolamine (aPE) [7] and phosphatidylserine (aPS) [8] in SLE patients [9,10]. Moreover, their relation to cardiovascular disorders such as ischemic stroke [11,12,13] and myocardial infarction [14] was also proved. The current study presents a novel approach as it was aimed at the complex evaluation of an association between the presence of aPE and aPS and various clinical manifestations in the course of the disease including early atherosclerotic changes and cardiovascular manifestations, microcirculatory abnormalities, thromboembolic complications, vasculitis and renal involvement as well as atherosclerotic risk factors, serological profile and applied treatment in SLE patients.

## 2. Material and Methods

### 2.1. Patients and Control Subjects

The study was approved by local ethical committee (KB-0012/11/13) and all subjects participating gave written informed consent.

The study was performed in 93 Caucasian SLE patients (81 women and 12 men) in age ranged from 19 to 74 years (mean 44.5 years) chosen in consecutive manner for studies at Department of Rheumatology, Internal Medicine, Geriatrics and Clinical Immunology Pomeranian Medical University in Szczecin. The diagnosis was established according to American College of Rheumatology Classification criteria [15]. The course of the disease ranged from 1 to 30 years (median 7.0 years). The activity of SLE was assessed on the basis of Systemic Lupus Erythematosus Activity Index (SLEDAI) [16]. The coexistence of APS was diagnosed on the basis of Sydney criteria [4]. Furthermore, other clinical manifestations were taken into consideration: cardiovascular disorders (coronary artery disease and/or myocardial infarction, left ventricular function abnormalities, hypokinesis, relaxation abnormalities, cerebral stroke and/or transient ischemic attacks), renal involvement, vasculitis and Raynaud’s phenomenon. The treatment data were collected. The control group consisted of 30 healthy volunteers age and gender matched with the patient group.

### 2.2. Imaging Diagnostics

All SLE patients and matched controls underwent noninvasive imaging investigations. All of the analyses were performed with HDI 3500 (ATL) using a 5–12 MHz linear transducer by the same ultrasonographist, who had 20 years of experience in vascular ultrasound.

The subclinical atherosclerosis was identified as an increase in cIMT. cIMT measurements were performed with B-mode ultrasound in common carotid artery, bifurcation and internal carotid artery on the right and left sides according to procedures previously described [17].

As a result of high variability of this parameter in populations [18,19] we established the normal and pathological range of cIMT values on the basis of measurements in the controls. Values ≤ 0.65 mm were considered as the reference range. Values between 0.66 mm and 0.86 mm were considered as a moderate cIMT and values above 0.86 mm as a high cIMT.

The B-mode ultrasound was also used as a screening for atherosclerotic plaque presence in carotid and lower extremities arteries (iliac, common femoral, deep femoral, superficial femoral, popliteal and tibial arteries) [20].

Ankle-brachial index was assessed using Doppler ultrasonography and calculated as a ratio of systolic pressure measured in the posterior tibial and dorsal arteries of both feet to the systolic pressure in the brachial artery. The abnormal values were considered at ABI < 1.0 [21].

High resistance index (HRI) was measured with duplex Doppler method under standardized conditions according to the procedure previously described [22]. Doppler spectral waveform was obtained bilaterally from the external iliac, common femoral, superficial femoral and popliteal arteries. The calculation of HRI was based on spectral waveforms obtained from popliteal arteries.

### 2.3. Classical Risk Factors for Atherosclerosis and Laboratory Tests

The National Cholesterol Educational Program Adult Treatment Panel III criteria were used to identify risk factors for atherosclerosis [23]. Weight and height were measured to calculate body mass index (BMI). We recorded data concerning diabetes, smoking habits (current smoking or smoking in the past, the duration of smoking in years, number of cigarettes per day), oral contraceptive application and positive family history for cardiovascular diseases.

Blood was taken after at least 8 h of fasting for an assessment of: uric acid (modified Trinder *assay based on the* methods *of* Trivedi and Kabasakalian), homocysteine (fluorescent polarization immunoassay), C-reactive protein (CRP) (turbidimetric nephelometry), erythrocyte sedimentation rate (ESR) (Westergren method), total cholesterol (enzymatic, based on the formulation of Allain, et al. and the modification of Roeschlau), direct low density lipoproteins (LDL), direct high density lipoproteins (HDL) cholesterol (enzymatic, colorimetric), direct triglycerides (enzymatic, colorimetric), glucose (hexokinase-mediated reaction) and fibrinogen (Clauss method). We also analyzed lipid indices including Castelli index classified as low (<4.5), moderate (≤4.5 to <7.0) and high (≥7.0); Kannel index classified as low (<3.0) and high (≥3.0); TG/HDL-cholesterol ratio classified as elevated score ≥ 3.0 [24,25,26]. Urinary status was evaluated by urine test strips (Siemens Multistix) and urinary sediment. Proteinuria was estimated by the 24-h urine albumin excretion (g/day). Renal function was assessed by plasma creatinine concentration (μmol/L) (kinetic colorimetric assay based on Jaffe’s reaction) and by the estimated Glomerular Filtration Rate (eGFR), as determined by the Modification of Diet in Renal Disease (MDRD) Study equation. To define kidney involvement proteinuria ≥ 0.5 g/day or eGFR < 50% were considered as pathological values.

### 2.4. Serological Diagnostics

The profile of aPL consisted of classic antibodies included in APS criteria—aCL and aβ2-GPI determined with enzyme linked immunosorbent assay (ELISA) method (EUROIMMUN AG Medizinische Labordiagnstika tests, Germany) and LA tested with coagulological methods according to International Society of Thrombosis and Haemostasis criteria [4]. Additionally, ELISA method was used for detection of aPT (AESKU.LAB DIAGNOSTIKA, Germany) and anti-oxidized low density lipoprotein antibodies (aoxLDL) (IMTEC Immunodiagnostika, Germany). The determinations of aPS (IgG and IgM isotypes) and aPE (IgG and IgM isotypes) were performed with ELISA method using Demeditec (Germany) and The Binding Site (UK) tests, respectively.

IgG antinuclear antibodies (ANA) were assessed on HEp-2 cell line contaminated by CVCL_0030 cervical adenocarcinoma human HeLa using indirect immunofluorescence assay (IIFA) and with monospecific tests performed with ELISA method for the detection of anti-double stranded DNA (adsDNA), anti-nucleosome (aNuA), anti-Sm, anti-SS-A/Ro, anti-SS-B/La, anti-ribosomal P protein, anti-histone (aHistone) and anti-U1-RNP antibodies (EUROIMMUN AG Medizinische Labordiagnostika tests, Germany).

The profile of anti-neutrophil cytoplasmic antibodies (ANCA) included screening IIFA for cytoplasmic (C-ANCA) and perinuclear (P-ANCA) staining patterns and monospecific tests performed with ELISA method for detection of anti-proteinase 3, anti-myeloperoxidase, anti-lactoferrin, anti-cathepsin G, aEla and anti-BPI antibodies (EUROIMMUN AG Medizinische Labordiagnostika tests, Germany).

The anti-endothelial cell antibodies were tested with human umbilical vein endothelial cells using IIFA method (EUROIMMUN AG Medizinische Labordiagnostika tests, Germany).

### 2.5. Statistical Analysis

All continuous variables were checked for equality distribution with Kolmogorov-Smirnov test. Data are described as mean ± standard deviation and median (Q1, Q3). A comparison of continuous variables was performed by Mann-Whitney and Student’s t-test. For categorical variables, differences were assessed by logistic regression model. In logistic regression model probability (*p*) was assessed by a chi-square testing or Fisher’s exact test. Results were shown as a *p*, odds ratio (OR) and 95% Confidence Interval (95%CI). Findings were considered statistically significant at *p* < 0.05. All of the statistical analyses were performed with STATISTICA version 8.0, StatSoft Inc., Tulsa, OK, USA.

## 3. Results

The detailed demographic, clinical, laboratory and therapeutic characteristics of the patient group and the controls are presented in Table 1.

The analysis of the occurrence of aPE and aPS showed the total presence of these autoantibodies (IgG or IgM isotype) in 18.3% and 12.9% of SLE patients, respectively (*p* = 0.044 and *p* = 0.038). The specific comparison of the prevalence of these antibodies in SLE patients and in the controls is demonstrated in Table 2.

aPS of IgG isotype was the sole aPL in one patient but no clinical associations were found (*p* > 0.05). On the other hand, aPE IgG were the sole aPL in four patients (4.3%). One patient presented a spectrum of vascular involvement, including high values of carotid intima-media thickness (cIMT), vasculitis, Raynaud’s phenomenon and thromboembolic complications. In one patient, high values of cIMT, and in two renal involvement was confirmed. None of these patients fulfilled the APS criteria (Table 3).

There was also analyzed the relationship between aPS and aPE and APS as well as other aPL in SLE patients. aPS of both isotypes was significantly associated with APS and all classical aPL including aCL, aβ2-GPI and LA. aPE IgM showed significant correlation with APS, aCL and aβ2-GPI. On the other hand, aPE IgG presented significant association only with aCL IgG (Table 4).

From the whole spectrum of analyzed organ disorders, only microcirculatory abnormalities were associated with aPS. In SLE patients with aPS (both IgG and IgM isotypes) the risk of Raynaud’s phenomenon development was significantly higher (OR = 4.5; 95%CI: 1.26–16.11, *p* = 0.021).

Further analysis demonstrated important relationship between aPE and the risk of selected clinical manifestations. The presence of aPE IgG was significantly associated with the risk of kidney involvement (OR = 3.5; 95%CI: 1.01–12.18, *p* = 0.049) and the occurrence of selected antibodies including adsDNA (OR = 5.10; 95%CI: 1.28–20.32, *p* = 0.021), aNuA (OR = 3.53; 95%CI: 1.02–12.26, *p* = 0.047), ANCA (OR = 5.10; 95%CI: 1.28–20.32, *p* = 0.021) and anti-elastase (aEla) (OR = 5.32; 95%CI: 1.06–26.74, *p* = 0.042) (Table 5).

On the other hand, in patients with aPE IgM we confirmed notably increased risk of cerebral stroke (OR = 20.4; 95%CI: 1.01–413.06, *p* = 0.050) (Table 5).

Moreover, the presence of aPE of both isotypes (IgG and IgM) was significantly associated with increased risk of thickening of carotid intima-media (OR = 4.2; 95%CI: 1.06–16.26, *p* = 0.041) in SLE patients. There was also an important association between aPE IgG and/or IgM and smoking status: the long duration of regular smoking (OR = 4.3; 95%CI: 1.25–14.66, *p* = 0.021) and the high number (≥20) of cigarettes/day (OR = 8.8; 95%CI: 1.53–50.80, *p* = 0.015) (Table 5).

Furthermore, the presence of aPS IgM was related to left ventricular posterior wall thickening in echocardiographic examination (OR = 5.28; 95%CI: 0.80–34.67; *p* = 0.083) and thromboembolic disorders (OR = 4.44; 95%CI: 0.82–24.04; *p* = 0.084) as well as Raynaud’s phenomenon (OR = 2.87; 95%CI: 0.93–8.78; *p* = 0.066). However, these findings were only on the border of statistical significance.

We did not find any significant relationship between aPS and aPE and other analyzed clinical manifestations, as well as applied treatment (*p* > 0.05).

We also compared SLE patients with low activity (≤6) with the patients presenting moderate (7–12) and high (>12) activities of the disease according to the SLEDAI scale. We confirmed in patients with SLEDAI score > 6 significantly more frequent presence of renal involvement, Raynaud’s phenomenon and coexistence of APS as well as occurrence of aPL, adsDNA and aHistone (Table 6). Moreover, the detailed analysis of patients with the highest SLEDAI score (>12) showed in this patient group importantly higher incidences of relaxation disorders, thromboembolic abnormalities, vasculitis and APS coexistence (myocardial infarction and renal involvement were on the border of statistical significance). They also presented wide spectrum of autoantibodies including aPL, ANA (adsDNA, aHistone, aNucleosome, aSm) and ANCA. Additionally, the assessment of lipid profile showed on the border of statistical significance higher frequency of elevated Castelli index comparing to SLE patients with low and moderate SLEDAI scores (Table 7).

We took into account the classical atherosclerotic risk factors and found significant associations between cardiovascular involvement in SLE patients and hypertension (OR = 4.28; 95%CI: 1.22–15.03; *p* = 0.023), overweight and obesity (OR = 3.71; 95%CI: 1.31–10.53; *p* = 0.014), tabagism (OR = 4.48; 95%CI: 1.44–13.92; *p* = 0.010), LDL-cholesterol (OR = 5.01; 95%CI: 1.49–16.82; *p* = 0.009), age ≥ 45 in males and ≥ 55 in females (OR = 5.83; 95%CI: 1.21–28.13; *p* = 0.028), uric acid (OR = 4.35; 95%CI: 1.25–15.17; *p* = 0.021), diabetes (OR = 7.08; 95%CI: 1.28–39.19; *p* = 0.25) and homocysteine (OR = 4.63; 95%CI: 1.43–15.05; *p* = 0.011).

The complex analysis of lipid profile in lupus patients confirmed significant statistical correlations between Castelli index, Kannel index and TG/HDL-cholesterol ratio and atherosclerotic changes in lower extremities arteries as well as thromboembolic disorders (Table 8).

## 4. Discussion

The presence of aPL as well as the coexistence of APS are quite common features in the course of SLE. The clinical significance of different aPL, which are not included in the APS criteria, has been intensively studied for many years. Our earlier reports demonstrated the usefulness of anti-prothrombin antibodies (aPT) in the diagnosis of APS in SLE patients and the highest specificity showed aPT IgG (95.12%). Additionally, aPT IgG were significantly associated with selected central nervous system manifestations, and aPT IgM importantly influenced the risk of development of cardiac complications and mononeuropathy. Interestingly, aPT IgA were significantly related to pleurisy and leucopenia, but they did not associate with the coexistence of APS [27]. Our study, which focused on atherosclerotic changes development in SLE patients, disclosed a significant relationship between aPT IgA and an increase in cIMT, which was confirmed by multivariate backward stepwise analysis [28].

These findings provoked further research on the clinical utility of other aPL. The current study was aimed at the evaluation of the association between the presence of aPS and aPE and the risk of selected organ manifestations development including cardiovascular disorders, renal involvement and microcirculation abnormalities in the course of SLE. In addition, we assessed connection of aPS and aPE with APS, selected autoantibodies as well as atherosclerotic risk factors providing the complex analysis of the clinical significance of these autoantibodies in SLE patients.

We found aPS in 12.9% and aPE in 18.3% of lupus patients. In one patient, aPS IgG were the sole aPL but we did not find any clinical association. In contrast, four patients with aPE IgG as the sole aPL showed selected vascular manifestations including thromboembolic disorders, vasculitis, atherosclerotic changes and Raynaud’s phenomenon. Our findings are supported by some clinical studies confirming aPE as the only aPL especially in patients suffering from thrombotic disease [29,30]. They were also found the sole antibodies in SLE patients with pulmonary embolism [31], thrombosis [32], valvulopathy and livedo reticularis [9]. Moreover, the report on Sneddon’s syndrome confirmed presence of aPE in 54% of patients and they were considered a major factor of microthrombosis of small arteries in this population [33]. *Sanmarco* et al. [34,35] stated that aPE might be defined a biological variant of APS in patients with unexplained thrombosis. In current study we demonstrated that there is no correlation between aPE IgG and APS as well as majority of other aPL in SLE patients who presented several vascular manifestations as described above, which may support this theory. It might be also hypothesized that patients with aPE, especially of the IgG isotype, should be considered at general vascular risk and screened, apart from thrombosis, for atherosclerotic disorders, microcirculation abnormalities and vasculitis. However, this issue needs further analysis in prospective studies on larger patient groups to fully explain the potential role of aPE in vascular injury development. The significant association between aPS of both isotypes IgG and IgM and APS in SLE patients documented in our study is also well confirmed by other reports [9,10,36,37].

In the current study, we found an important correlation between aPE and kidney injury. In accordance, the report by *Fialova* et al. [38] on SLE patients with renal involvement confirmed the high frequency of noncardiolipin antibodies including aPE, aPS and anti-phosphatidylinositol antibodies in those patients and underlined the importance of various aPL in selecting patients with lupus nephritis. In addition, the study performed in pediatric SLE patients with biopsy confirmed lupus nephritis showed that patients with aPL more frequently presented class IV nephritis, microthrombi in small arterioles and intra-glomerular microthrombosis, higher creatinine levels and proteinuria in comparison with aPL negative patients [39]. Our study also showed significant association between aPE IgG, adsDNA and aNuA. adsDNA are markers of SLE and their role in lupus nephritis development and assessment of activity of the disease have been confirmed by many researchers. *Gamal* et al. [40] showed in Egyptian population the importantly higher frequency of APS in SLE patients positive for adsDNA. However, there was no correlation between adsDNA and particular aPL in the analyzed patient group. *Loizou* et al. [41] found the presence of aCL in conjunction with raised levels of adsDNA and anti-C1q antibodies was highly specific for lupus nephritis. The investigation performed in female SLE patients also revealed that adsDNA were remarkably higher in patients with aPL, but there was no relation between adsDNA and renal injury in the patient group [42]. In this regard our results together with previous observations on the role of IL-23 in lupus nephritis development [43] might be helpful in understanding pathomechanisms underlying renal injury development in SLE patients and the potential role of aPL in this process. Additionally, significant relationship between aPE IgG and adsDNA might be helpful in the activity of the disease assessment and prediction of exacerbation. However, it needs to be confirmed in further prospective studies. Furthermore, an interesting finding of a correlation between aPE and ANCA was reported in our study. There are little data available in the literature on the coexistence of aPL and ANCA. A report by *Yoo* et al. [44] showed that persistent aPL at diagnosis did affect thrombotic events during follow-up in ANCA-associated vasculitides (AAV) patients suggesting that there are overlapping pathomechanisms triggered by different types of autoantibodies which may affect the vessel wall. On the one hand, aPL provokes a thrombogenic state via various mechanisms including complement and platelets activation, adhesion molecules expression or tissue factor up-regulation. On the other hand, the severity of AAV is considered to be associated with platelet activation markers and coagulation or fibrinolysis indices. Thus, authors state that all kinds of aPL can increase the risk of thrombotic complication in AAV patients at a high rate and preventive antiaggregating or anticoagulation procedures should be taken into consideration in all patients with the concomitant presence of aPL at diagnosis or during follow-up.

Our study also revealed a significant relationship between aPE and cerebrovascular events in SLE patients and remains in accordance with other data. An investigation performed in 185 patients, including SLE patients, suffering from stroke showed aPE in 35% of patients. Furthermore, the presence of aPE was the most frequent finding in patients who were suspected to have an associated APS [45]. The next study on young non-SLE patients without obvious causes of arterial thromboembolism who underwent ischemic cerebrovascular incidences also demonstrated the presence of wide profile of non-cardiolipin aPL, including aPE. However, the frequency of aPE was lower: 10.4% [46]. This difference may suggest that aPE is a better serologic marker of cerebrovascular events in patients suffering from systemic connective tissue diseases than in the general population. An interesting case report illustrated the novel findings of aPE in the cerebral spinal fluid of a 15-year-old patient with a documented ischemic stroke suggesting a possibility of an intrathecal production of aPL in the course of central nervous system disorders [47]. However, this observation needs to be confirmed by further investigations.

Our research showed a significant association between aPE and early atherosclerotic lesions development presented as the increase in cIMT. These data were confirmed by the analysis of the clinical features of 20 patients with aPE only, among whom 17 had symptoms potentially related to APS. The authors pointed out a relationship between aPE and arteriosclerosis with peripheral arteriopathy [48]. Furthermore, all findings with a prelation of aPE to cerebrovascular involvement, very often based on atherosclerotic origin, indirectly support these observations. A significant association between cIMT and the risk of ischemic stroke also has been well-documented [25,49,50]. Of note, the latest observations on atherosclerosis in SLE patients with coexisting APS showed that atherosclerotic plaques are infiltrating by T helper (Th) cells secreting IL-17 and interferon (IFN)-γ in response to β2-GPI and suggest that β2-GPI drives a local Th17/Th1 inflammatory response, which can be responsible for plaque instability and rupture, leading to atherothrombosis [51]. Our previous study also supports these data. We have documented a significant association between IL-23, aPLs including aPE IgG and atherosclerotic plaque development in SLE patients [43]. As IL-23 plays a central role in inflammation including the induction of Th17 cells [52,53] these observations might contribute to better understanding of the pathomechanism of SLE and its complications such as atherosclerosis and cardiovascular disease development. Further studies are necessary to fully elucidate this issue.

Additionally, the analysis of aPE and classical atherosclerotic risk factors showed significant correlation with smoking status in SLE patients. aPE was elevated in patients with long history of regular smoking (current and past) and those who smoked more than 20 cigarettes per day. Tabagism is considered to be an important pathogenic factor of systemic autoimmune rheumatic diseases. The risk of developing SLE is also related to the average number of cigarettes smoked per day, cigarette-years of smoking, fraction smoked per cigarette and degree of smoke inhalation. Moreover, smoking status is associated with selected clinical manifestations such as skin involvement, lupus nephritis and thrombotic events as well as pathogenic antibodies production including adsDNA and aPL [54]. A prospective cohort study in SLE patients revealed that smoking was associated with aCL, aβ2-GPI and LA. Of note, aPL found in patients who did not smoke regularly was not associated with vascular events. Undoubtedly, the relation between smoking and aPL is important in vascular, including atherothrombotic, lesions development in SLE patients [55,56]. The causal relationship between these two factors is still unclear. Some authors suggest the “two-hit hypothesis” to explain why aPL alone does not always initiate clinical manifestations of APS [57]. Further studies are necessary to elucidate this problem.

We also noted in our study a remarkable association between aPS and Raynaud’s phenomenon. There was also trend towards a higher risk of this complication in patients with the presence of aPE, however, these findings were only on the border of statistical significance. The contrary results were shown in other study focused on identification of rare aPL in SLE patients. Admittedly, their influence on the increased frequency of the clinical symptoms and complications of SLE and APS was confirmed, but a relationship between aPS and Raynaud’s phenomenon was not found [10]. On the other hand, an impact of aPL on microcirculatory abnormalities in SLE patients was shown in a few earlier studies suggesting a possible link between these antibodies and endothelial damage. Patients with the presence of aCL more often developed Raynaud’s phenomenon and livedo reticularis [58], as well as changes in nailfold capillaroscopy [59].

Furthermore, we disclosed the trend towards left ventricular posterior wall thickening in SLE patients with aPS. The association between aPL, including aPS, anti-phosphatidylinositol as well as anti-phosphatidic acid antibodies and cardiac impairment in lupus patients was reported indicating an adjunctive role of aPL in these complications developments [60].

Finally, the cardiovascular risk in lupus patients is a very complex phenomenon. Classical risk factors as well as factors associated with the disease and immune system dysregulation are involved in vascular impairment development. Our study showed significant impact of classical atherosclerotic risk factors, especially dyslipidemia, and activity of the disease on vascular involvement in SLE patients which was also confirmed by other studies [24,25,26].

To summarize, the limitation of our study is a small number of patients presenting high activity of the disease with multiple organ involvement. Nevertheless, the novel approach based on complex analysis of vascular involvement in the course of SLE enabled us to show a noticeable relationship between analyzed autoantibodies, especially aPE, and the risk of vascular manifestations development both in macro- and micro-circulation. Of note, aPE also might serve as a marker of activity of the disease as well as renal involvement in SLE patients. Significant correlation with smoking and early atherosclerotic changes supports in the identification of patients at risk of cardiovascular complications. Moreover, both aPS and aPE increase the risk of development of clinical manifestations of APS in SLE patients. Especially, aPE might expand the diagnostic potential of serological markers in APS diagnosis as they are associated with the risk of thrombotic complications occurrence in SLE patients negative for the aPL included into APS criteria. Further studies with larger number of patients are needed to fully explain the role of rare aPL in pathogenesis of vascular changes development in SLE patients and to establish their diagnostic capability and managing strategy.

## 5. Conclusions

This study shows that SLE patients with aPS and aPE are at risk of vascular involvement. Especially in case of absence classical serological markers of APS the presence of aPE may significantly increase the risk of thrombotic complications development in lupus patients. Additionally, aPE might serve as a marker of disease activity and risk of renal injury development in this patient group.

The general cardiovascular risk assessment in SLE patients should be also based on classical atherosclerotic markers, with a special focus on lipid profile evaluated using lipid indices which can help to identify patients at the highest risk to implement appropriate preventive, diagnostic and therapeutic procedures.

## Figures and Tables

**Table 1:** Clinical and laboratory characteristics of systemic lupus erythematosus patients and healthy controls.

Assessed Parameters	Systemic Lupus Erythematosus Patients	Healthy Controls
	n = 93	n = 30
	Mean ± SD	Mean ± SD
	Median (Q1, Q3)	Median (Q1, Q3)
Age (years)	44.5 ± 13.5	43.5 ± 14.1
Sex	F-81 M-12	F-24 M-6
Disease duration (years)	7.0 (4.0, 12.0)	-
SLEDAI: low, n (%)	52 (55.9)	-
medium, n (%)	32 (34.4)	-
high, n (%)	9 (9.7)	-
APS, n (%)	31 (33.3)	-
renal involvement, n (%)	24 (25.8)	-
cerebrovascular manifestations:		
TIA, n (%)	2 (2.2)	-
stroke, n (%)	10 (10.8)	-
cardiovascular manifestations:		
CAD, n (%)	10 (10.8)	-
MI, n (%)	4 (4.3)	-
Left ventricular function abnormalities, n (%)	11 (11.8)	-
Hypokinesis, n (%)	10 (10.8)	-
Relaxation abnormalities, n (%)	10 (10.8)	-
Raynaud’s phenomenon, n (%)	27 (29.0)	-
vasculitis, n (%)	14 (15.1)	-
Thromboembolic disorders, n (%)	19 (20.4)	-
cIMT (mm)	0.70 (0.65, 080)	0.60 (0.60, 0.68)
ABI right	1.08 (1.03, 1.16)	1.12 (1.07, 1.24)
ABI left	1.08 (1.02, 1.16)	1.14 (1.08, 1.22)
HRI right	0.314 (0.252, 0.390)	0.388 (0.365, 0.429)
HRI left	0.328 (0.217, 0.394)	0.439 (0.379, 0.471)
Plaques n (%):		
cca	3 (3.2)	0 (0.0)
bulb	18 (19.4)	0 (0.0)
ica	2 (2.2)	0 (0.0)
carotid arteries	21 (22.6)	0 (0.0)
iliaca	8 (8.6)	0 (0.0)
cfa	19 (20.4)	0 (0.0)
dfa	0 (0.0)	0 (0.0)
sfa	10 (10.8)	0 (0.0)
popla	4 (4.3)	0 (0.0)
pta	0 (0.0)	0 (0.0)
Lower extremities arteries	27 (29.0)	0 (0.0)
Hypertension, n (%)	37 (39.8)	2 (6.7)
BMI	25.0 ± 4.9	24.2 ± 3.5
Diabetes, n (%)	11 (11.8)	0 (0.0)
Smoking habits, n (%)	32 (34.4)	12 (40.0)
Oral contraceptive use, n (%)	5/81 (6.2)	6/24 (25.0)
Family history of cardiovascular disease, n (%)	4 (4.3)	4 (13.3)
Total cholesterol (mg/dL)	218.5 ± 59.8	228.4 ± 39.8
LDL-cholesterol (mg/dL)	129.1 ± 47.7	138.5 ± 33.6
HDL-cholesterol (mg/dL)	58.9 ± 24.5	62.4 ± 12.0
Triglycerides (mg/dL)	150.0 ± 91.2	138.9 ± 70.6
Castelli index	4.03 ± 1.61	3.78 ± 0.88
Kannel index	2.41 ± 1.09	2.28 ± 0.64
TG/HDL-cholesterol ratio	3.38 ± 6.08	2.42 ± 1.82
CRP (mg/L)	2.6 (1.2, 6.1)	0.0 (0.0, 1.0)
ESR (mm/h)	22.0 (12.0, 45.0)	9.0 (2.0, 16.0)
Fibrinogen (mg/dL)	316.0 (271.0, 375.0)	278.0 (250.0, 338.0)
Homocysteine (mol/L)	13.9 (11.0, 18.2)	6.6 (5.4, 8.0)
Uric acid (mg/dL)	4.6 (3.9, 5.9)	4.1 (3.4, 5.3)
Antinuclear antibodies IgG	73 (78.5)	2 (6.7)
anti-double stranded DNA IgG	39 (41.9)	0 (0.0)
anti-nucleosome IgG	30 (32.3)	0 (0.0)
anti-Sm IgG	4 (4.3)	0 (0.0)
anti-SS-A/Ro IgG	41 (44.1)	0 (0.0)
anti-SS-B/La IgG	14 (15.1)	0 (0.0)
anti-rybosomal P protein IgG	6 (6.5)	0 (0.0)
anti-histon IgG	20 (21.5)	0 (0.0)
anti-U1-RNP IgG	19 (20.4)	0 (0.0)
anti-cardiolipin IgG	33 (35.5)	0 (0.0)
anti-cardiolipin IgM	19 (20.4)	1 (3.3)
anti-cardiolipin IgA	23 (24.7)	1 (3.3)
anti-beta2-glycoprotein I IgG	7 (7.5)	0 (0.0)
anti-beta2-glycoprotein I IgM	22 (23.7)	0 (0.0)
anti-beta2-glycoprotein I IgA	24 (25.8)	1 (3.3)
anti-oxidized low density lipoprotein IgG	43 (46.2)	1 (3.3)
anti-oxidized low density lipoprotein IgM	67 (72.0)	2 (6.7)
anti-prothrombin IgG	10 (10.8)	0 (0.0)
anti-prothrombin IgM	11 (11.8)	1 (3.3)
anti-prothrombin IgA	11 (11.8)	1 (3.3)
anti-phosphatidylserine IgG	10 (10.8)	0 (0.0)
anti-phosphatidylserine IgM	7 (7.5)	0 (0.0)
anti-phosphatidylethanolamine IgG	12 (12.9)	0 (0.0)
anti-phosphatidylethanolamine IgM	6 (6.5)	1 (3.3)
lupus anticoagulant	14 (15.1)	0 (0.0)
anti-neutrophil cytoplasmic antibodies IgG	39 (41.9)	2 (6.7)
anti-proteinase 3 IgG	0 (0.0)	0 (0.0)
anti-myeloperoxidase IgG	9 (9.7)	0 (0.0)
anti-lactoferrin IgG	13 (13.9)	0 (0.0)
anti-elastase IgG	9 (9.7)	0 (0.0)
anti-BPI IgG	1 (1.1)	0 (0.0)
anti-cathepsin G IgG	11 (11.8)	0 (0.0)
anti-endothelial cell antibodies IgG	42 (45.2)	2 (6.7)
Immunosupprissive treatment:		
Encorton	86 (92.5)	-
Endoxan	47 (50.5)	-
Azathioprine	43 (46.2)	-
Chlorambucil	4 (4.3)	-
Cyclosporine A	2 (2.2)	-
Methotrexate	5 (5.4)	-
Chloroquine	53 (57.0)	-

SLEDAI—systemic lupus erythematosus disease activity index; APS—antiphospholipid syndrome; TIA—transient ischemic attacks; CAD—coronary artery disease; MI—myocardial infarction, cIMT—carotid intima-media thickness; ABI—ankle-brachial index; cca—common carotid arteries; ica—internal carotid arteries; iliaca—iliac arteries; cfa—common femoral arteries; dfa—deep femoral arteries; sfa—superficial femoral arteries; popla—popliteal arteries; pta—posterior tibial arteries, BMI—body mass index, LDL—low density lipoprotein; HDL—high density lipoprotein, CRP—C-reactive protein; ESR—erythrocyte sedimentation rate, BPI—bactericidal/permeability-increasing protein. –: the parameter was not assessed in healthy controls.

**Table 2:** Presence of anti-phosphatidylserine and anti-phosphatidylethanolamine antibodies in patients with systemic lupus erythematosus in comparison with the controls.

Parameter		Patients with SLE Number (%)	Control Group Number (%)	*p*
aPS IgG	No	83 (89.3)	30 (100.0)	0.061
	Yes	10 (10.8)	0 (0.0)	
aPS IgM	No	86 (92.5)	30 (100.0)	0.122
	Yes	7 (7.5)	0 (0.0)	
aPS IgG/IgM	No	81 (87.1)	30 (100.0)	0.038
	Yes	12 (12.9)	0 (0.0)	
aPE IgG	No	81 (87.1)	30 (100.0)	0.038
	Yes	12 (12.9)	0 (0.0)	
aPE IgM	No	87 (93.5)	29 (96.7)	0.522
	Yes	6 (6.5)	1 (3.3)	
aPE IgG/IgM	No	76 (81.7)	29 (96.7)	0.044
	Yes	17 (18.3)	1 (3.3)	

aPS—anti-phosphatidylserine antibodies, aPE—anti-phosphatidylethanolamine antibodies, SLE—systemic lupus erythematosus.

**Table 3:** Clinical characteristics of patients with systemic lupus erythematosus with anti-phosphatidylethanolamine antibodies of IgG isotype as a sole antiphospholipid antibody.

Patient	cIMT	lupus Nephritis	Vasculitis	Raynaud’s Phenomenon	Thromboembolic Complications	Antiphospholipid Syndrome
1	+	−	+	+	+	−
2	+	−	−	−	−	−
3	−	+	−	−	−	−
4	−	+	−	−	−	−

aIMT—carotid intima-media thickness; +: the presence of a clinical manifestation; −: the absence of a clinical manifestation.

**Table 4:** The relation of anti-phosphatidylserine and anti-phosphatidylethanolamine antiantibodies to antiphospholipid syndrome and other antiphospholipid antibodies in systemic lupus erythematosus patients.

Anti-Phosphtidylserine Antibodies IgG			
Covariate	OR*	95%CI	*p*
Antiphospholipid Syndrome	12.42	2.32–66.57	0.003
aCL IgG	18.67	2.21–158.04	0.007
aCL IgM	15.87	2.99–84.32	0.001
LA	72.48	9.74–539.38	0.000
aβ2-GPI IgG	97.72	9.12–1047.47	0.000
aβ2-GPI IgM	11.44	2.35–55.80	0.003
aPT IgG	4.25	0.84–21.56	0.080
aPT IgM	4.77	0.92–24.74	0.063
**Anti-Phosphtidylserine Antibodies IgM**			
Antiphospholipid Syndrome	5.76	1.05–31.65	0.044
aCL IgG	16.37	1.78–150.81	0.014
aCL IgM	43.17	10.33–1969.67	0.000
LA	23.50	3.80–145.37	0.001
aβ2-GPI IgG	9.40	1.13–77.82	0.038
aβ2-GPI IgM	33.13	8.04–1512.92	0.000
aPT IgG	4.05	0.66–24.89	0.130
aPT IgM	7.18	1.36–37.97	0.020
**Anti-Phosphtidylethanolamine Antibodies IgG**			
	OR*	95%CI	*p*
Antiphospholipid Syndrome	2.59	0.69–9.70	0.156
aCL IgG	4.48	1.23–16.27	0.023
aCL IgM	0.41	0.05–3.64	0.422
LA	0.97	0.17–5.42	0.972
aβ2-GPI IgG	1.67	0.27–10.44	0.584
aβ2-GPI IgM	0.86	0.16–4.69	0.859
aPT IgG	7.67	1.43–41.29	0.018
aPT IgM	0.75	0.08–7.11	0.799
**Anti-Phosphtidylethanolamine Antibodies IgM**			
Antiphospholipid syndrome	11.81	1.32–106.78	0.028
aCL IgG	5.18	0.84–31.99	0.077
aCL IgM	34.15	8.20–1590.65	0.000
LA	3.41	0.55–21.27	0.189
aβ2-GPI IgG	19.54	1.57–242.59	0.021
aβ2-GPI IgM	19.61	2.12–181.88	0.009
aPT IgG	5.58	0.84–37.22	0.076
aPT IgM	22.93	3.48–151.04	0.001

*OR adjusted for age and gender. aCL—anti-cardiolipin antibodies, aβ2-GPI—anti-beta2 –glycoprotein I antibodies, LA—lupus anticoagulant, aPT—antiprothrombin antibodies

**Table 5:** A logistic regression model of the OR of the presence of anti-phosphatidylethanolamine antibodies in systemic lupus erythematosus patients.

Covariates	aPE IgG		aPE IgM		aPE IgG or IgM	
	OR* (95%CI)	*p*	OR* (95%CI)	*p*	OR* (95%CI)	*p*
Renal involvement	3.50 (1.01–12.18)	0.049	0.44 (0.01–1.77)	0.135	1.28 (0.39–4.21)	0.680
Cerebral stroke	0.48 (0.01–1.95)	0.153	20.38 (1.01–413.06)	0.050	0.97 (0.18–5.05)	0.967
Moderately thickened intima-media	3.13 (0.77–12.77)	0.112	2.18 (0.19–24.51)	0.527	4.16 (1.06–16.26)	0.041
Raynaud’s phenomenon	3.50 (0.91–13.45)	0.069	2.61 (0.49–13.94)	0.261	2.87 (0.93–8.78)	0.066
The duration of regular smoking 1–19 years	5.40 (1.33–21.90)	0.018	3.18 (0.46–21.83)	0.240	4.28 (1.25–14.66)	0.021
The number of cigarettes/day ≥ 20	12.56 (0.96–165.03)	0.054	7.43 (1.12–49.30)	0.038	8.80 (1.53–50.80)	0.015
adsDNA	5.10 (1.28–20.32)	0.021	0.29 (0.03–2.69)	0.275	1.37 (0.45–4.12)	0.577
aNuA	3.53 (1.02–12.26)	0.047	1.34 (0.21–8.51)	0.754	2.95 (1.00–8.65)	0.049
ANCA	5.10 (1.28–20.32)	0.021	1.68 (0.31–9.22)	0.550	3.14 (1.05–9.43)	0.041
aEla	5.32 (1.06–26.74)	0.042	0.90 (0.03–6.10)	0.407	4.37 (1.03–18.47)	0.045

*OR adjusted for age and gender. aPE—anti-phosphatidylethanolamine antibodies, adsDNA—anti-double stranded DNA antibodies, aNuA—anti-nucleosome antibodies, ANCA—anti-neutrophil cytoplasmic antibodies, aEla—anti-elastase antibodies

**Table 6:** The comparison of systemic lupus erythematosus patients presenting low activity of the disease with patients presenting medium and high activity according to the SLEDAI scale.

Parameter		SLE Patients with Disease Activity According to SLEDAI Scale ≤ 6 (%)	SLE Patients with Disease Activity According to SLEDAI Scale > 6 (%)	OR	95%CI	*p*
Raynaud’s phenomenon	No	42 (80.8)	24 (58.5)	2.91	1.17–7.24	0.021
	Yes	10 (19.2)	17 (41.5)			
Renal involvement	No	44 (84.6)	25 (61.0)	3.79	1.45–9.90	0.006
	Yes	8 (15.4)	16 (39.0)			
APS	No	40 (76.9)	22 (53.7)	2.75	1.19–6.37	0.018
	Yes	12 (23.1)	19 (46.3)			
aCL IgG	No	40 (76.9)	20 (48.8)	3.01	1.30–6.95	0.010
	Yes	12 (23.1)	21 (51.2)			
aCL IgG/IgM	No	36 (69.2)	17 (41.5)	3.04	1.35–6.85	0.007
	Yes	16 (30.8)	24 (58.5)			
aCL/LA	No	35 (67.3)	17 (41.5)	3.08	1.37–6.92	0.007
	Yes	17 (32.7)	24 (58.5)			
aPT IgG	No	49 (94.2)	34 (82.9)	4.07	1.01–16.39	0.048
	Yes	3 (5.8)	7 (17.1)			
adsDNA	No	34 (65.4)	20 (48.8)	2.32	1.04–5.21	0.041
	Yes	18 (34.6)	21 (51.2)			
aHistone	No	49 (94.2)	30 (73.2)	4.78	1.69–13.56	0.003
	Yes	3 (5.8)	11 (26.8)			

SLE—systemic lupus erythematosus, SLEDAI—systemic lupus erythematosus disease activity index, APS—antiphospholipid syndrome; aCL—anti-cardiolipin antibodies, LA—lupus anticoagulant, aPT—antiprothrombin antibodies, adsDNS—anti-double stranded DNA antibodies.

**Table 7:** The comparison of systemic lupus erythematosus patients presenting low and medium activity of the disease with patients presenting high activity according to the SLEDAI scale.

Parameter		SLE Patients with Disease Activity according to SLEDAI Scale ≤ 12 (%)	SLE Patients with Disease Activity according to SLEDAI Scale > 12 (%)	OR	95%CI	*p*
Myocardial infarction	No	82 (97.6)	7 (77.8)	6.59	0.97–44.79	0.052
	Yes	2 (2.4)	2 (22.2)			
Relaxation abnormalities	No	76 (90.5)	7 (77.7)	5.25	1.01–27.39	0.049
	Yes	8 (9.5)	2 (22.2)			
Renal involvement	No	64 (76.2)	5 (55.6)	3.00	0.83–10.86	0.094
	Yes	20 (23.8)	4 (44.4)			
Thromboembolic disorders	No	69 (82.1)	5 (55.6)	3.68	1.00–13.47	0.049
	Yes	15 (17.9)	4 (44.4)			
Vasculitis	No	74 (88.1)	5 (55.6)	4.16	1.04–16.53	0.043
	Yes	10 (11.9)	4 (44.4)			
APS	No	59 (70.2)	3 (33.3)	6.42	1.58–26.05	0.009
	Yes	25 (29.8)	6 (66.7)			
aCL IgG	No	57 (67.9)	3 (33.3)	3.80	1.03–14.02	0.045
	Yes	27 (32.1)	6 (66.7)			
aCL IgM	No	69 (82.1)	5 (55.6)	4.28	1.15–15.84	0.030
	Yes	15 (17.9)	4 (44.4)			
aCL IgG/IgM	No	51 (60.7)	2 (22.2)	7.33	1.50–35.90	0.014
	Yes	33 (39.3)	7 (77.8)			
aPT IgG	No	78 (92.9)	5 (55.6)	11.81	2.78–50.18	0.001
	Yes	6 (7.1)	4 (44.4)			
aPT IgM	No	77 (91.7)	5 (55.6)	5.93	1.42–24.69	0.014
	Yes	7 (8.3)	4 (44.4)			
adsDNA	No	53 (63.1)	1 (11.1)	19.68	2.41–160.78	0.005
	Yes	31 (36.9)	8 (88.9)			
aNuA	No	60 (71.4)	3 (33.3)	4.44	1.20–16.46	0.026
	Yes	24 (28.6)	6 (66.7)			
aSm	No	82 (97.6)	8 (88.9)	11.13	1.92–64.42	0.007
	Yes	2 (2.4)	1 (11.1)			
aHistone	No	74 (88.1)	5 (55.6)	3.68	1.00–13.47	0.049
	Yes	10 (11.9)	4 (44.4)			
aRibosomal P protein	No	80 (95.2)	7 (77.8)	8.16	1.55–43.02	0.013
	Yes	4 (4.8)	2 (22.2)			
ANCA	No	52 (61.9)	2 (22.2)	7.33	1.50–35.90	0.014
	Yes	32 (38.1)	7 (77.8)			
Castelli index	No	63 (75.0)	4 (44.4)	3.55	0.00–12.73	0.052
	Yes	21 (25.0)	5 (55.6)			

SLE—systemic lupus erythematosus, SLEDAI—systemic lupus erythematosus disease activity index, APS—antiphospholipid syndrome; aCL—anti-cardiolipin antibodies, LA—lupus anticoagulant, aPT—antiprothrombin antibodies, adsDNA—anti-double stranded DNA antibodies, aNuA—anti-nucleosome antibodies, aSm—anti-Smith, ANCA—anti-neutrophil cytoplasmic antibodies.

**Table 8:** A logistic regression model of the OR of the presence of high values of cardiometabolic lipid indices in systemic lupus erythematosus patients.

Covariates	Castelli Index		Kannel Index		TG/HDL-Cholesterol	
	OR* (95%CI)	*p*	OR* (95%CI)	*p*	OR* (95%CI)	*p*
Plaques in iliac arteries	4.86 (1.08–21.89)	0.039	3.70 (0.85–16.11)	0.081	1.69 (0.40–7.17)	0.480
Plaques in left superficial femoral arteries	7.40 (1.35–40.64)	0.021	5.00 (1.03–24.18)	0.045	4.49 (0.03–24.37)	0.082
Thromboembolic disorders	3.85 (1.41–10.51)	0.008	3.30 (1.18–9.25)	0.023	1.63 (0.62–4.30)	0.323

*OR adjusted for age and gender. TG—triglicerides, HDL—high density lipoproteins.

## Data Availability

The data used to support the findings of this study are available from the corresponding author upon request.

## References

[1] Aringer M., Costenbader C., Daikh D., Brinks R., Mosca M., Ramsey-Goldman R., Smolen J.S., Wofsy D., Boumpas D.T., Kamen D.L. (2019). 2019 European League Against Rheumatism/American College of Rheumatology Classification Criteria for Systemic Lupus Erythematosus. Arthritis Rheum..

[2] Molokhia M., McKeigue P. (2006). Systemic lupus erythematosus: Genes versus environment in high risk populations. Lupus.

[3] Criswell L.A. (2008). The genetic contribution to systemic lupus erythematosus. Bull. NYU Hosp. Jt. Dis..

[4] Miyakis S., Lockshin M.D., Atsumi T., Branch D.W., Brey R.L., Cervera R., Derksen R.H., DE Groot P.G., Koike T., Meroni P.L. (2006). International consensus statement on an update of the classification criteria for definite antiphospholipid syndrome (APS). J. Thromb. Haemost..

[5] Maneta-Peyet L., Previsani C., Sultan Y., Bezian J.H., Cassagne C. (1991). Autoantibodies against all the phospholipids: A comparative systematic study with systemic lupus erythematosus and healthy sera. Eur. J. Clin. Chem. Clin. Biochem..

[6] Toschi V., Motta A., Castelli C., Gibelli S., Cimminiello C., Molaro G.L., Gibelli A. (1993). Prevalence and clinical significance of antiphospholipid antibodies to noncardiolipin antigens in systemic lupus erythematosus. Haemostasis.

[7] Mcintyre J.A., Wagenknecht D.R. (2000). Anti-phosphatidylethanolamine (aPE) antibodies: The survey. J. Autoimmun..

[8] Bertolaccini M.L., Hughes G.R. (2006). Antiphospholipid antibody testing: Which are most useful for diagnosis?. Rheum. Dis. Clin. N. Am..

[9] Balada E., Ordi-Ros J., Paredes F., Villarreal J., Mauri M., Vilardell-Tarréset M. (2001). Antiphosphatidylethanolamine antibodies contribute to the diagnosis of antiphospholipid syndrome in patients with systemic lupus erythematosus. Scand. J. Rheumatol..

[10] Szodoray P., Tarr T., Tumpek J., Kappelmayer J., Lakos G., Poór G., Szegedi G., Kiss E. (2009). Identification of rare anti-phospholipid/protein co-factor autoantibodies in patients with systemic lupus erythematosus. Autoimmunity.

[11] Kahles T., Humpich M., Steinmetz H., Sitzer M., Lindhoff-Last E. (2005). Phosphatidylserine IgG and beta-2-glycoprotein I IgA antibodies may be a risk factor for ischaemic stroke. Rheumatology.

[12] Saidi S., Mahjoub T., Almawi W.Y. (2009). Lupus anticoagulants and anti-phospholipid antibodies as risk factors for a first episode of ischemic stroke. J. Thromb. Haemost..

[13] Okuma H., Kitagawa Y., Takagi S. (2010). Investigation of antiphosphatidylserine antibody and antiphosphatidylinositol antibody in ischemic stroke patients. Clin. Dev. Immunol..

[14] Jansen N.L., Snell J.R., Moy J.N. (1996). Myocardial infarction as the presenting manifestation of systemic lupus erythematosus with antiphosphatidylserine antibodies. Ann. Allergy Asthma Immunol..

[15] Hochberg M.C. (1997). Updating the American College of Rheumatology revised criteria for the classification of systemic lupus erythematosus. Arthritis Rheum..

[16] Bombardier C., Gladman D.D., Urowitz M.B., Caron D., Chang C.H. (1992). Derivation of the SLEDAI. A disease activity index for lupus patients. The Committee on Prognosis Studies in SLE. Arthritis Rheum..

[17] Howard G., Sharrett A.R., Heiss G., Evans G.W., Chambless L.E., Riley W.A., Burke G.L. (1993). Carotid artery intimal—medial thickness distribution in general populations as evaluated by B—mode ultrasound. Stroke.

[18] Ebrahim S., Papacosta O., Whincup P., Wannamethee G., Walker M., Nicolaides A.N., Dhanjil S., Griffin M., Belcaro G., Rumley A. (1999). Carotid plaque, intima media thickness, cardiovascular risk factors, and prevalent cardiovascular disease in men and women. The British Regional Heart Study. Stroke.

[19] Homma S., Hirose N., Ishida H., Ishii T., Araki G. (2001). Carotid plaque and intima-media thickness assessed by B-mode ultrasonography in subjects ranging from young adults to centenarians. Stroke.

[20] Vlachoyiannopoulos P.G., Kanellopoulos P.G., Ioannidis J.P.A., Tektonidou M.G., Mastorakou I., Moutsopouloset H.M. (2003). Atherosclerosis in premenopausal women with antiphospholipid syndrome and systemic lupus erythematosus: A controlled study. Rheumatology.

[21] Sacks D., Bakal C.W., Beatty P.T., Becker G.J., Cardella J.F., Raabe R.D., Wiener H.M., Lewis C.A. (2002). Position statement on the use of the ankle-brachial index in the evaluation of patients with peripheral vascular disease. J. Vasc. Interv. Radiol..

[22] Walecka A., Sawicki M., Brzosko M., Ostanek L., Fischer K., Kordowski J. (2004). Value of high resistance index—HRI calculated from Doppler spectrum of popliteal arteries in patients with systemic lupus erythematosus (SLE). Med. Sci. Monit. Int. Med. J. Exp. Clin. Res..

[23] Brewer B.H. (2003). New features of the national cholesterol education program adult treatment panel III lipid-lowering guidelines. Clin. Cardiol..

[24] Pesqueda-Cendejas K., Parra-Rojas I., Mora-Garcia P.E., Montoya-Buelna M., Ruiz-Ballesteros A.I., Meza-Meza M.R., Campos-López B., Rivera-Escoto M., Vizmanos-Lamotte B., Cerpa-Crus S. (2022). CRP serum levels are associated with high cardiometabolic risk and clinical disease activity in systemic lupus erythematosus patients. J. Clin. Med..

[25] de Jesús Batún Garrido J.A., Alba H.A.R., Núñez É.H., Olán F. (2016). Dyslipidemia and atherogenic risk in patients with systemic lupus erythematosus. Med. Clin..

[26] Campos-López B., Meza-Meza M.R., Parra-Rojas I., Ruiz-Ballesteros A.I., Vizmanos-Lamotte B., Muñoz-Valle J.F., Montoya-Buelna M., Cerpa-Cruz S., Bernal-Hernández L.E., De la Cruz-Mosso U. (2021). Association of cardiometabolic risk status with clinical activity and damage in systemic lupus erythematosus patients: A cross-sectional study. Clin. Immunol..

[27] Ostanek L., Brzosko M., Fischer K. (2005). Usefulness of different antiphospholipid antibodies in the assessment of the secondary antiphospholipid syndrome in lupus erythematosus patients—Review and own experiences. Reumatologia.

[28] Fischer K., Brzosko M. (2009). Diagnosis of early atherosclerotic lesions, and selected atherosclerotic risk factors, in patients with systemic lupus erythematosus. Pol. Arch. Med. Wewn..

[29] Bérard M., Chantome R., Marcelli A., Boffa M.C. (1996). Antiphosphatidylethanolamine antibodies as the only antiphospholipid antibodies. I. Association with thrombosis and vascular cutaneous diseases. J. Rheumatol..

[30] Sanmarco M., Alessi M.C., Harle J.R., Sapin C., Aillaud M.F., Gentile S., Juhan-Vague I., Weiller P.J. (2001). Antibodies to phosphatidylethanolamine as the only antiphospholipid antibodies found in patients with unexplained thromboses. Thromb. Haemost..

[31] Karmochkine M., Cacoub P., Piette J.C., Godeau P., Boffa M.C. (1992). Antiphosphatidylethanolamine antibody as the sole antiphospholipid antibody in systemic lupus erythematosus with thrombosis. Clin. Exp. Rheumatol..

[32] Karmochkine M., Berard M., Piette J.C., Cacoub P., Aillaud M.F., Harlé J.R., Godeau P., Boffa M.C., Harlet J.R. (1993). Antiphosphatidylethanolamine antibodies in systemic lupus erythematosus. Lupus.

[33] Kalashnikova L.A., Aleksandrova E.N., Novikov A.A., Dobrynina L.A., Nasonov E.L., Sergeeva E.V., Berkovskiĭ A.L. (2005). Anti-phosphatidylethanolamine antibodies in patients with Sneddon’s syndrome. Klin. Med..

[34] Sanmarco M., Gayet S., Alessi M.C., Audrain M., de Maistre E., Gris J.C., de Groot P.G., Hachulla E., Harlé J.R., Sié P. (2007). Antiphosphatidylethanolamine antibodies are associated with an increased odds ratio for thrombosis. A multicenter study with the participation of the European Forum on antiphospholipid antibodies. Thromb. Haemost..

[35] Sanmarco M., Boffa M.C. (2009). Antiphosphatidylethanolamine antibodies and the antiphospholipid syndrome. Lupus.

[36] Lopez L., Dier K.J., Lopez D., Merrill J.T., Fink C.A. (2004). Anti-β_2_-Glycoprotein I and antiphosphatidylserine antibodies are predictors of arterial thrombosis in patients with antiphospholipid syndrome. Am. J. Clin. Pathol..

[37] Khogeer H., Alfattani A., Al Kaff M., Al Shehri T., Khojah O., Owaidah T. (2015). Antiphosphatidylserine antibodies as diagnostic indicators of antiphospholipid syndrome. Lupus.

[38] Fialova L., Zima T., Tesař V., Mikulíková L., Malbohan I.M., Merta M., Certíková V. (2003). Antiphospholipid antibodies in patients with lupus nephritis. Ren. Fail..

[39] Al-Mayouf S.M., AlSaleem A., Al-Hussain T., Al Sonbul A., AlMana H. (2015). The impact of antiphospholipid antibodies in children with lupus nephritis. Int. J. Pediatr. Adolesc. Med..

[40] Gamal S.M., Fawzy S., Siam I. (2013). Does anti-DNA positivity increase the incidence of secondary antiphospholipid syndrome in lupus patients?. Egypt. Rheumatol..

[41] Loizou S., Samarkos M., Norsworthy P.J., Cazabon J.K., Walport M.J., Davies K.A. (2000). Significance of anticardiolipin and anti-beta(2)-glycoprotein I antibodies in lupus nephritis. Rheumatology.

[42] Gheita T.A., Abaza N.M., Hammam N., Mohamed A.A.A., El-Gazzar I.I., Eissa A.H. (2018). Anti-dsDNA titre in female systemic lupus erythematosus patients: Relation to disease manifestations, damage and antiphospholipid antibodies. Lupus.

[43] Fischer K., Przepiera-Będzak H., Sawicki M., Walecka A., Brzosko I., Brzosko M. (2017). Serum interleukin-23 in Polish patients with systemic lupus erythematosus: Association with lupus nephritis, obesity, and peripheral vascular disease. Mediat. Inflamm..

[44] Yoo J., Ahn S.S., Jung S.M., Song J.J., Park Y.B., Lee S.W. (2019). Persistent antiphospholipid antibodies are associated with thrombotic events in ANCA-associated vasculitis: A retrospective monocentric study. Nefrologia.

[45] Gonzales-Portillo F., Mcityre J.A., Wagenknecht D.R., Williams L.S., Bruno A., Biller J. (2001). Spectrum of antiphospholipid antibodies (aPL) in patients with cerebrovascular disease. J. Stroke Cerebrovasc. Dis..

[46] Toschi V., Motta A., Castelli C., Paracchini M.L., Zerbi D., Gibelli A. (1998). High prevalence of antiphosphatidylinositol antibodies in young patients with cerebral ischemia of undetermined cause. Stroke.

[47] Sokol D.K., Mcintyre J.A., Short R., Gutt J., Wagenknecht D.R., Biller J., Garg B. (2000). Henoch-Schönlein purpura and stroke: Antiphosphatidylethanolamine antibody (aPE) in CSF and serum. Neurology.

[48] Desauw C., Hachulla E., Boumbar Y., Bouroz-Joly J., Ponard D., Arvieux J., Dubucquoi S., Fauchais A.L., Hatron P.Y., Devulder B. (2002). Antiphospholipid syndrome with only antiphosphatidylethanolamine antibodies: Report of 20 cases. Rev. Med. Interne.

[49] Chambless L.E., Folsom A.R., Clegg L.X., Sharrett A.R., Shahar E., Nieto F.J., Rosamond W.D., Evans G. (2000). Carotid wall thickness is predictive of incident clinical stroke. The Atherosclerosis Risk in Communities (ARIC) Study. Am. J. Epidemiol..

[50] Touboul P.J., Labreuche J., Vicaut E., Amarenco P. (2005). Carotid intima-media thickness, plaques, and Framingham risk score as independent determinants of stroke risk. Stroke.

[51] Benagiano M., Borghi M.O., Romagnoli J., Mahler M., Della Bella C., Grassi A., Capitani N., Emmi G., Troilo A., Silvestri E. (2019). Interleukin-17/interleukin-21 and interferon-γ producing T cells specific for β2 Glycoprotein I in atherosclerosis inflammation of systemic lupus erythematosus patients with antiphospholipid syndrome. Haematologica.

[52] Parham C., Chirica M., Timans J., Vaisberg E., Travis M., Cheung J., Pflanz S., Zhang R., Singh K.P., Vegaet F. (2002). A receptor for the heterodimeric cytokine IL-23 is composed of IL-12Rbeta1 and a novel cytokine receptor subunit, IL-23R. J. Immunol..

[53] Duvallet E., Semerano L., Assier E., Falgarone G., Boissier M.C. (2011). Interleukin 23 a key cytokine in inflammatory diseases. Ann. Med..

[54] Perricone C., Versini M., Ben-Ami D., Gertel S., Watad A., Segel M.J., Ceccarelli F., Conti F., Cantarini L., Bogdanos D.P. (2016). Smoke and autoimmunity: The fire behind the disease. Autoimmun. Rev..

[55] Gustafsson J.T., Simard J.F., Gunnarsson I., Elvin K., Lundberg I.E., Hansson L.O., Larsson A., Svenungsson E. (2012). Risk factors for cardiovascular mortality in patients with systemic lupus erythematosus, a prospective cohort study. Arthritis Res. Ther..

[56] Gustafsson J.T., Gunnarsson I., Källberg H., Pettersson S., Zickert A., Vikerfors A., Möller S., Rönnelid J., Elvin K., Svenungsson E. (2015). Cigarette smoking, antiphospholipid antibodies and vascular events in systemic lupus erythematosus. Ann. Rheum. Dis..

[57] Meroni P.L., Ronda N., De Angelis V., Grossi C., Raschi E., Borghi M.O. (2007). Role of anti-beta2 glycoprotein I antibodies in antiphospholipid syndrome: In vitro and in vivo studies. Clin. Rev. Allergy Immunol..

[58] Buchanan R.R., Wardlaw J.R., Riglar A.G., Littlejohn G.O., Miller M.H. (1989). Antiphospholipid antibodies in the connective tissue diseases: Their relation to the antiphospholipid syndrome and forme fruste disease. J. Rheumatol..

[59] Bongard O., Boumaneaux H., Miescher P.A., De Moerlooseet P. (1995). Association of anticardiolipin antibodies and abnormal nailfold capillaroscopy in patients with systemic lupus erythematosus. Lupus.

[60] Amoroso A., Cacciapaglia F., De Castro S., Battagliese A., Coppolino G., Galluzzo S., Vadacca M., Afeltra A. (2006). The adjunctive role of antiphospholipid antibodies in systemic lupus erythematosus cardiac involvement. Clin. Exp. Rheumatol..

